# Supplementary material for: An extensible vector toolkit and parts library for advanced engineering of plant genomes
Source: Plant Genome. 2023 Mar 9;16(2):e20312. doi: 10.1002/tpg2.20312 (PMC12807070; doi:10.1002/tpg2.20312)
Supplement: Supplementary file 1 [file TPG2-16-e20312-s004.pdf]

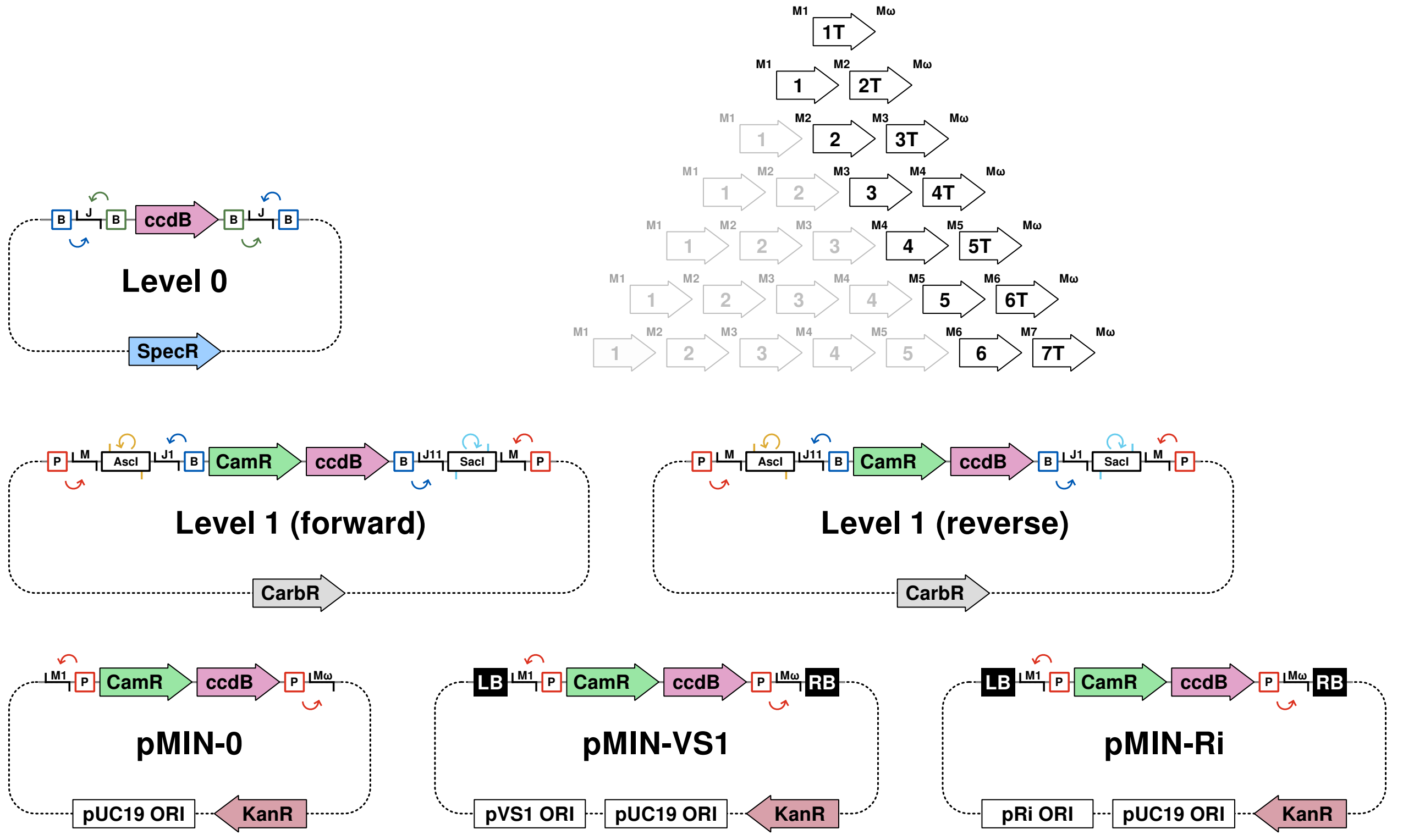

Figure S1: Destination vectors for hierarchical assembly. Destination vectors, from Level 0 through Level 2, use *ccdB* counterselection. Level 1 destination vectors are available for each position in the 7-module circuit with both forward and reverse orientation Phytobrick entry, and feature AscI and SacI sites for traditional cloning in addition to BsaI Golden Gate entry.

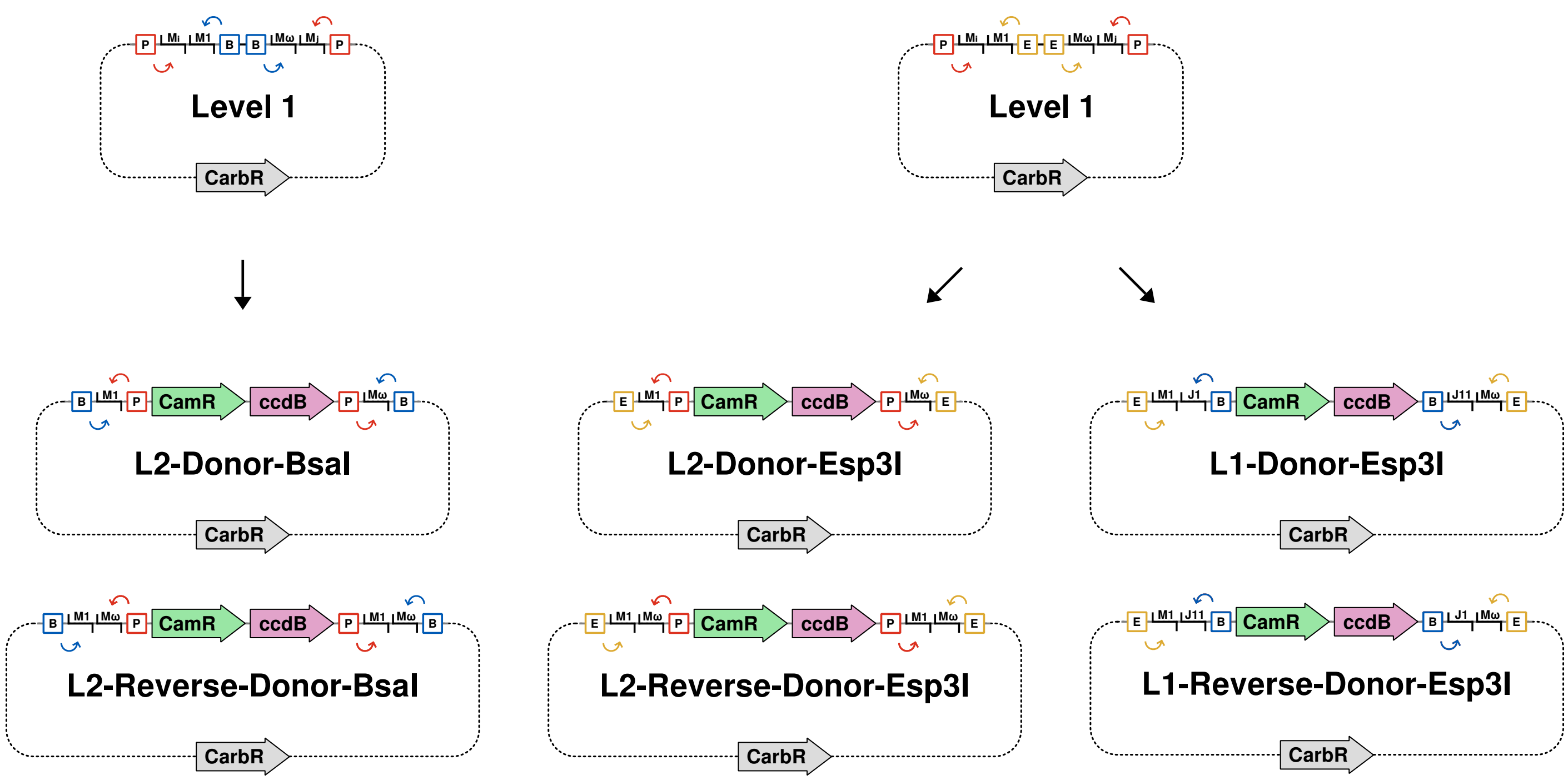

Figure S2: Vector sets for modular expansion system. Level 1 expansion modules contain internal Esp3I or BsaI linkers. BsaI linkers can be used to introduce L2 donors, and Esp3I linkers can be used to introduce L1 or L2 donors.
